# Driving contradictions: behaviors and attitudes regarding handheld and hands-free cellphone use while driving among young drivers

**DOI:** 10.1186/s40621-021-00312-2

**Published:** 2021-06-01

**Authors:** Lucas M. Neuroth, Dylan Galos, Li Li, Songzhu Zhao, Motao Zhu

**Affiliations:** 1 The Center for Injury Research and Policy, Abigail Wexner Research Institute at Nationwide Children’s Hospital, 575 Children’s Crossroad, Columbus, OH 43215 USA; 2 Office of Statewide Health Improvement Initiatives, Community Health Division, Minnesota Department of Health, 85 East 7th Place, Suite 220, PO Box 64882, Saint Paul, MN 55164 USA; 3 Division of Epidemiology, College of Public Health, The Ohio State University, Cunz Hall 1841 Neil Avenue, Columbus, OH 43210 USA; 4 Department of Biomedical Informatics, Center for Biostatistics, The Ohio State University, 320 Lincoln Tower, 1800 Cannon Drive, Columbus, OH 43210 USA; 5 Department of Pediatrics, College of Medicine, The Ohio State University, 370 W. 9th Avenue, Columbus, OH 43210 USA

**Keywords:** Distracted driving, Crashes, Traffic, Text messaging, Adolescent, Young adult

## Abstract

**Background:**

Cellphone use while driving (CUWD) is a frequent source of distraction for young drivers. These distractions commonly lead to motor vehicle crashes and, in some cases, death. Crash risk differs depending on if the driver is engaging in handheld or hands-free CUWD. This pilot study sought to investigate the differences between handheld versus hands-free CUWD behaviors in younger drivers and the attitudes and social norms that inform them.

**Methods:**

Young drivers (mean age: 19.6 years, standard deviation: 0.8 years) were recruited from a large Midwestern city in the United States as part of a pilot study. The 62 enrolled drivers (51 females, 43 non-Hispanic white) completed an online survey measuring behavioral frequencies, attitudes, and social norms regarding talking on the phone, sending messages, and reading messages. These cross-sectional data were then categorized and used for a descriptive analysis.

**Results:**

The majority of young drivers reported participating in some form of handheld CUWD, with reading messages being the most popular (95%). Only 43% of participants used hands-free technology for sending messages and 30% for reading messages, while half reported not using the technology at all. Whereas handheld messaging behaviors were viewed negatively by the participants, they were unsure of the impact on their driving ability and the legality surrounding hands-free messaging behaviors.

**Conclusions:**

Handheld CUWD behaviors were more popular among young drivers compared to hands-free CUWD. Further, even though young drivers understood handheld cellphone use while driving is unsafe, they engaged in it anyway. The findings of this pilot study highlight the importance of better educational initiatives and optimizing hands-free interventions for young driver use cases.

## Background

Within the United States, the leading cause of death for those aged 15 to 24 years is unintentional injury (National Center for Injury Prevention and Control 2020). Among unintentional fatalities in this age group, 52% were due to motor vehicle crashes (MVCs), accounting for 6308 deaths in 2018 (National Center for Injury Prevention and Control 2020). A significant contributor of MVCs is distracted driving, with younger drivers being more likely to be involved in fatal distracted driving-related crashes (National Center for Statistics and Analysis 2020). One frequent source of driver distraction is cellphone use while driving (CUWD).

CUWD can be separated into handheld and hands-free variants. Handheld CUWD increases the risk of MVCs by competing for the driver’s attention and removing their hands from the steering wheel (Coben and Zhu 2013). It is associated with longer detection and avoidance times for roadway stimuli, as well as with compensatory driving behavior, both of which function to increase crash risk (Caird et al. 2018). The risk of MVCs is highest when the driver is engaged in handheld texting, emailing or other forms of messaging (Caird et al. 2014; Fitch et al. 2013). This is particularly worrying with regard to younger drivers, as 71 to 91% report participating in some form of handheld messaging while driving (Cazzulino et al. 2014).

To address the risks surrounding handheld CUWD, both automotive and cellphone manufacturers have developed various hands-free technologies. Cellphone-based hands-free technologies include speakerphone, voice commands, and headphone use, while vehicle-based hands-free solutions include auxiliary cable inputs, infotainment integration, and Bluetooth connections. Further, each of these technologies differ in their implementation, with some functioning purely without driver input (e.g. asking one’s phone to place a call), and others requiring visual-motor input (e.g. looking at the center console to see who is calling and tapping a screen to answer), potentially impacting levels of driver distraction. Comparing hands-free CUWD tasks to driving without cellphone use, researchers found no significant difference in the odds of safety critical events (Fitch et al. 2013). When investigating infotainment system use in drivers aged 21 to 36, researchers found the time to complete various hands-free tasks differed across task type, with calling taking the least amount of time compared to texting and navigation entry (Cooper et al. 2019).

Carter et al. (2014) report 92% of novice drivers engage in some form of distracted driving, similar to Hill et al. (2015)‘s findings regarding texting and calling while driving among college students. While these and national-level estimates exist for the prevalence of CUWD (Rudisill and Zhu 2015), they do not investigate the potential differences between handheld and hands-free CUWD. Similarly, while many studies have documented the attitudes and social norms surrounding CUWD for these young drivers (Atchley et al. 2012; Carter et al. 2014; Rudisill and Zhu 2015), there is little research regarding how they may differ across handheld and hands-free use, an important factor to consider when developing effective interventions for distracted driving.

We aim to address this research gap by describing the frequency of handheld and hands-free CUWD among young drivers and investigating the attitudes and social norms that may inform these behaviors using self-reported data from a pilot study.

## Methods

### Participants and procedure

This pilot study was conducted within a large Midwestern city in the United States, from 2017 to 2019. Participants were recruited for the pilot study though social media advertising campaigns and a local university’s study search program. Following initial contact, 390 potential participants were screened for age, smartphone ownership, a full driver’s license, and access to a car they drove the majority of the week. After screening, 106 potential participants were ruled ineligible due to one (*n* = 73) or more (*n* = 33) factors, with the most common reasons being age – 43% of the excluded participants were 21 years or older (not young drivers) – and driving less than 3 days a week (33%).

Of the 284 eligible potential participants remaining, 62 were selected for enrollment on a rolling, first-come first-served basis. Each of the selected participants were then described the study procedures by a trained research assistant and, after signing a written consent form, enrolled in the study. Most participants were female (*n* = 51, 82%) and of non-Hispanic White race (*n* = 43, 69%), with an average age of 19.6 years (range: 18–20 years, standard deviation: 0.8 years). Once enrolled, participants were administered an online survey which included instruments to assess frequency, attitudes, and social norms regarding talking on the phone, sending messages, and reading messages. Participants were awarded $50 for completing this survey and additional study objectives during their initial appointment. This pilot study was approved by our Institutional Review Board.

### Measures

#### Definitions of handheld and hands-free CUWD

Handheld CUWD was defined as the participant physically holding the cellphone while engaging in an activity. Hands-free CUWD was defined as the participant utilizing devices such as Bluetooth and speakerphone while engaging in an activity, ensuring no physical interaction with the cellphone.

#### CUWD behaviors

Frequencies of talking on the phone, sending messages, and reading messages were measured as a proportion of trips driven 30 days prior to survey administration. Response options included “never,” “approximately (10, 30, 50, 70, 90) percent of all my trips,” and “every trip,” with hands-free behaviors having an additional response option: “I don’t use this technology.” Frequencies were then categorized as “greater than 50% of trips,” “less than or equal to 50% of trips,” or “never” to account the small sample size and facilitate comparisons across CUWD type.

#### Attitudes and social norms regarding CUWD

Participants were questioned about their attitudes relating to the impact of CUWD behaviors on the following three outcomes: being in a crash, getting a traffic ticket, and negatively affecting their driving ability. Similar to CUWD behaviors, response options for crash and attitude questions were recoded from seven-option Likert-type scales (extremely unlikely, unlikely, slightly unlikely, neutral/unsure, slightly likely, likely, extremely likely) to three-level categorical variables (unlikely, neutral, and likely). Driving ability was measured using a three-level scale (no, maybe, and yes).

To investigate the social norms surrounding CUWD, participants were questioned regarding the perceived acceptability of CUWD behaviors from the following three perspectives: those of their closest friends, those of their parent(s) or guardian(s), and their own, as passenger in someone else’s vehicle. Again, responses were recoded from a seven-option Likert-type scale (totally unacceptable, unacceptable, slightly unacceptable, neutral, slightly acceptable, acceptable, totally acceptable) to three-level categorical variables (unacceptable, neutral, and acceptable). Additionally, participants were questioned regarding the perceived legality of CUWD behaviors using a three-level scale (forbidden, not sure, and permitted).

#### Vehicle-specific data

The availability of in-vehicle hands-free cellphone technology was determined through the analysis of the owner’s manual for each participant’s vehicle, based on make, model, and model year data provided in the survey. As the participants were young drivers, they were assumed to have the base-trim of their vehicle, with variables coded for the presence of a Bluetooth system, cassette player, auxiliary cable input, and infotainment screen.

### Analytic method

This study is a descriptive analysis of a cross-sectional survey. Frequencies were tabulated for handheld and hands-free CUWD behaviors. Differences in hands-free CUWD behaviors by in-vehicle hands-free technology were assessed using the Wilcoxon rank-sum test, accounting for the ordinal nature in which hands-free CUWD frequencies were collected.

Additionally, participant attitudes and social norms were tabulated for each CUWD behavior, stratified by handheld versus hands-free use. As each participant’s answers regarding handheld and hands-free CUWD were correlated and on an ordinal scale, the Wilcoxon matched-pairs signed-rank test was used to assess differences in attitudes and social norms between handheld and hands-free CUWD (Rey and Neuhäuser 2011). All analyses were conducted using STATA 15.1, with statistical significance set at alpha = 0.05.

## Results

The majority of participants enrolled in this pilot study engaged in some form of handheld CUWD in the 30 days prior to taking the survey (Table 1). The most prevalent handheld CUWD behavior was reading messages (95%), followed by talking on the phone (86%), then sending messages (84%). Hands-free technology was commonly used by the participants when talking on the phone, with 71% of participants using it during at least one trip in the last 30 days. Only 43% of participants used hands-free technology for sending messages and 30% for reading messages, while half reported not using the technology for messaging at all.

**Table 1:** Frequencies of handheld vs. hands-free cellphone use while driving behaviors

N (%^**a**^)	> 50% of trips^**b**^	≤ 50% of trips	Never	I don't use this technology
**Handheld**^**c**^ **cellphone use**				
Talking on the phone	6 (10)	47 (76)	9 (14)	NA
Sending messages^d^	11 (18)	41 (66)	10 (16)	NA
Reading messages	14 (23)	45 (72)	3 (5)	NA
**Hands-free**^**e**^ **cellphone use**				
Talking on the phone	6 (10)	38 (61)	6 (10)	12 (19)
Sending messages	7 (11)	20 (32)	7 (11)	28 (45)
Reading messages	7 (11)	12 (19)	12 (19)	31 (50)

Abbreviation: *NA* Not applicable

^a^Row percentages may not sum to 100 due to rounding

^b^Participants were asked to estimate the proportion of trips in the 30 days prior to the baseline survey they conducted each of the presented behaviors

^c^Handheld use was defined as physically holding the phone

^d^Messages were defined as text or email messages

^e^Hands-free use was defined as using devices such as Bluetooth and speaker phone so there is no physical interaction between the driver and their phone when engaging in the aforementioned behaviors

Approximately 70% of participants had at least one form of in-vehicle hands-free technology, the most prevalent being auxiliary cable inputs (37% of all vehicles) and the least prevalent being Bluetooth hands-free systems (8% of all vehicles). The frequency of hands-free CUWD behaviors were not statistically significantly different in the presence or absence of an in-vehicle Bluetooth system, cassette player, auxiliary cable input, or infotainment screen (data not presented).

Participants did, however, differ significantly in their attitudes regarding each handheld and hands-free CUWD behavior (Table 2). When questioned about the likelihood of being in a crash, participants felt handheld CUWD was more likely to result in a crash compared to hands-free behaviors. For both handheld and hands-free CUWD, more participants felt messaging behaviors resulted in a higher crash risk than talking on the phone. Sending messages was perceived to be the riskiest CUWD behavior among study participants, 73% believed the handheld variant of this behavior was likely to result in a crash, while 32% believed the same for hands-free sending. Additionally, participants felt handheld messaging behaviors would make them more likely to receive a traffic ticket compared to handheld calling, while 81% believed that hands-free behaviors were unlikely to result in a traffic ticket.

**Table 2:** Attitudes toward handheld and hands-free cellphone use while driving behaviors

	**Handheld**^**a**^ **CUWD**^**b**^ **Behaviors**			**Hands-free**^**c**^ **CUWD Behaviors**			
**Attitudes**^**d**^ **N (%**^**e**^**)**	**Unlikely**	**Neutral**	**Likely**	**Unlikely**	**Neutral**	**Likely**	***P*-value**^**f**^
**Being in a crash**							
Talking on the phone	32 (52)	6 (10)	24 (39)	46 (74)	4 (7)	12 (19)	**0.0001**
Sending messages^g^	14 (23)	3 (5)	45 (73)	37 (60)	5 (8)	20 (32)	**< 0.0001**
Reading messages	16 (26)	3 (5)	43 (69)	38 (61)	8 (13)	16 (26)	**< 0.0001**
**Getting a traffic ticket**							
Talking on the phone	37 (60)	8 (13)	17 (27)	50 (81)	4 (7)	8 (13)	**< 0.0001**
Sending messages	21 (34)	1 (2)	40 (65)	50 (81)	4 (7)	8 (13)	**< 0.0001**
Reading messages	26 (42)	3 (5)	33 (53)	50 (81)	2 (3)	10 (16)	**< 0.0001**
	**No**	**Maybe**	**Yes**	**No**	**Maybe**	**Yes**	
**Negatively affect driving ability**							
Talking on the phone	13 (21)	15 (24)	34 (55)	35 (57)	18 (29)	9 (15)	0.0619
Sending messages	0 (0)	6 (10)	56 (90)	13 (21)	35 (57)	14 (23)	**0.0161**
Reading messages	3 (5)	16 (26)	43 (69)	17 (27)	32 (52)	13 (21)	0.4203

^a^Handheld use was defined as physically holding the phone

^b^Cellphone use while driving (CUWD)

^c^Hands-free use was defined as using devices such as Bluetooth and speaker phone so there is no physical interaction between the driver and their phone when engaging in the aforementioned behaviors

^d^Participants were questioned about their attitudes relating to the impact of cellphone use while driving (CUWD) behaviors on the following three outcomes: being in a crash, getting a traffic ticket, and negatively affecting their driving ability

^e^Row percentages may not sum to 100 due to rounding

^f^*P*-value from the Wilcoxon matched-pairs signed-rank test comparing each handheld CUWD behavior to its hands-free variant, bolded when < 0.05

^g^Messages were defined as text or email messages

Regarding the perceived impact of CUWD behaviors on their driving ability, all handheld behaviors had a higher proportion of participants expressing a negative impact on their driving ability compared to their hands-free variant. That said, responses did not differ significantly for talking on the phone (*p* = 0.0619), nor reading text messages (*p* = 0.4203). Participants did, however, differ significantly in their responses surrounding the negative impact of sending messages (*p* = 0.0161), with 90% of participants believing handheld message sending would negatively impact their driving compared to 50% stating hands-free sending may have a negative impact on their driving ability.

Statistically significant differences were also observed between handheld and hands-free CUWD behaviors for each of the presented social norms scenarios (Table 3). Participants uniformly found handheld messaging behaviors to be socially unacceptable. Perceived perceptions regarding handheld messaging from the perspective of a parent or guardian and from themselves as a passenger were similar, with approximately 90% of participants finding these behaviors to be socially unacceptable from the aforementioned points of view. Handheld talking was perceived as acceptable by close friends (56%) and themselves as a passenger (57%), but unacceptable from a parental point of view (61%).

**Table 3:** Social norms toward handheld and hands-free cellphone use while driving behaviors

	**Handheld**^**a**^ **CUWD**^**b**^ **Behaviors**			**Hands-free**^**c**^ **CUWD Behaviors**			
**Social norms**^**d**^ **N(%**^**e**^**)**	**Unacceptable**	**Neutral**	**Acceptable**	**Unacceptable**	**Neutral**	**Acceptable**	***P*-value**^**f**^
**Closest friends**							
Talking on the phone	21 (34)	6 (10)	35 (56)	4 (7)	1 (2)	57 (92)	**< 0.0001**
Sending messages^g^	46 (74)	1 (2)	15 (24)	15 (24)	6 (10)	41 (66)	**< 0.0001**
Reading messages	40 (65)	2 (3)	20 (32)	13 (21)	6 (10)	43 (69)	**< 0.0001**
**Parent or guardian**							
Talking on the phone	38 (61)	2 (3)	22 (35)	13 (21)	4 (7)	45 (73)	**< 0.0001**
Sending messages	59 (95)	0 (0)	3 (5)	28 (45)	7 (11)	27 (44)	**< 0.0001**
Reading messages	56 (90)	1 (2)	5 (8)	26 (42)	9 (15)	27 (44)	**< 0.0001**
**Self as passenger**							
Talking on the phone	24 (39)	3 (5)	35 (57)	7 (11)	2 (3)	53 (86)	**< 0.0001**
Sending messages	57 (92)	1 (2)	4 (7)	17 (27)	9 (15)	36 (58)	**< 0.0001**
Reading messages	50 (81)	3 (5)	9 (15)	16 (26)	8 (13)	38 (61)	**< 0.0001**
	**Forbidden**	**Not Sure**	**Permitted**	**Forbidden**	**Not Sure**	**Permitted**	
**Permitted by law**^**h**^							
Talking on the phone	26 (43)	21 (34)	14 (23)	2 (3)	14 (23)	46 (74)	NA
Sending messages	54 (87)	6 (10)	2 (3)	9 (14)	26 (42)	27 (44)	NA
Reading messages	49 (79)	11 (18)	2 (3)	8 (13)	23 (37)	31 (50)	NA

Abbreviation: *NA* Not applicable

^a^Handheld use was defined as physically holding the phone

^b^Cellphone use while driving (CUWD)

^c^Hands-free use was defined as using devices such as Bluetooth and speaker phone so there is no physical interaction between the driver and their phone when engaging in the aforementioned behaviors

^d^Participants were questioned about their perceived acceptability (social norms) of CUWD behaviors from three perspectives: those of their closest friends, those of their parent(s) or guardian(s), and their own, as passenger in someone else’s vehicle

^e^Row percentages may not sum to 100 due to rounding

^f^*P*-value from the Wilcoxon matched-pairs signed-rank test comparing each handheld CUWD behavior to its hands-free variant, bolded when < 0.05

^g^Messages were defined as text or email messages

^h^Participants were questioned with regard to the perceived permissibility of CUWD behaviors on a legislative level

Most of participants viewed hands-free CUWD calling as acceptable within each social norms scenario. Hands-free messaging behaviors were also viewed as acceptable, though they perceived parental opinion surrounding sending/reading messages as unacceptable. Lastly, while most participants understood handheld messaging behaviors were not permitted by law, they were uncertain regarding the legality of both handheld calling and hands-free messaging behaviors.

## Discussion

Within this sample of young drivers, the majority engaged in handheld CUWD behaviors including talking on the phone and sending/receiving messages. Approximately 90% of participants engaged in at least one form of CUWD, mirroring results reported by Hill et al. (2015) in their survey of college-age drivers. A similar proportion of participants engaged in handheld and hands-free talking on the phone, while less than half of the young drivers utilized hands-free messaging technology. Only 11% of young drivers report its use in more than half of their trips, indicating a clear difference in handheld versus hands-free messaging behaviors.

Participants clearly identified the risks associated with handheld messaging behavior, along with the benefits and social acceptability of using hands-free technology. The contrast between attitudes/social acceptability and behavior raises questions regarding why the participants rarely used hands-free messaging technology. One potential explanation for this discrepancy is that perceived parent and peer acceptability toward handheld and hands-free behavior have little impact on behavior change. Delgado et al. (2018) found that while over 90% of young drivers were at least somewhat willing to give up messaging while driving, peer and parental concern were the least likely to be effective in reducing messaging behaviors, corroborating this point.

Over two-thirds of participants had access to at least one form of in-vehicle hands-free technology. Analogue technology (auxiliary input/cassette deck) was more prevalent among the participants than digital (Bluetooth/infotainment system), though no significant difference in the frequency of hands-free CUWD was observed between the presence or absence of the four in-vehicle technologies evaluated. Given over 50% of participants were unsure if hands-free messaging behaviors would negatively impact their driving ability, it is possible that hands-free messaging technology is not yet an effective proxy for handheld use. Further research into various in-phone and in-vehicle hands-free technologies could be helpful in elucidating their infrequent use among younger drivers and their potential for driver distraction.

Additionally, approximately 40% of participants were unsure of the legal permissibility of hands-free messaging behaviors and handheld calling. In 2012, our state’s legislature banned handheld messaging while driving, followed by handheld calling in 2018. This recent shift towards stricter CUWD policy may have contributed to the participants’ uncertainty regarding the legality of these behaviors. While stricter CUWD policy has been associated with decreased texting while driving, the implementation of these policies, and their enforcement, vary nationwide (Rudisill and Zhu 2015; Rudisill and Zhu 2016). Clear education and consistent enforcement surrounding the legality of hands-free CUWD technologies may increase their use among young drivers while decreasing the prevalence and safety consequences of distracted driving.

This study is not without limitations. The study population was 82% female; a demographic found to be safer at driving compared to young males (Ouimet et al. 2015). Further, the sample size was relatively small from a single Midwestern city, which could impact generalizability. Lastly, all data were self-reported and social desirability bias may have influenced participant responses.

That said, this study features several strengths. It is among the first to report the differences in CUWD stratified by handheld and hands-free use. Additionally, it highlights that while teen drivers understand the risks associated with handheld messaging behaviors, they continue to engage in them. Given the pilot nature of this study, further research should be undertaken to investigate these findings in the context of a larger, representative sample of young drivers in the United States.

## Conclusions

This pilot study presents clear differences in the attitudes and social norms surrounding handheld and hands-free CUWD, as well as between participant’s attitudes/social norms and their behaviors. As CUWD continues to be addressed using technology and policy, it is imperative to consider both the effectiveness of these interventions and how to properly communicate them to young drivers.

## Data Availability

The STATA code used for this paper’s analyses, as well as the de-identified data, are available from the corresponding author upon request.

## Abbreviations

MVCs: Motor vehicle crashes
CUWD: Cellphone use while driving
